# No evidence for European bats serving as reservoir for Borna disease virus 1 or other known mammalian orthobornaviruses

**DOI:** 10.1186/s12985-020-1289-3

**Published:** 2020-01-30

**Authors:** Daniel Nobach, Christiane Herden

**Affiliations:** 1 Institute of Veterinary Pathology, Justus Liebig University, Giessen, Germany; 2 Center for Mind, Brain and Behavior, Justus Liebig University, Giessen, Germany

**Keywords:** Borna disease virus 1, Reservoir, Bats, Bavaria, Germany, Bornaviridae

## Abstract

**Background:**

The majority of emerging infectious diseases are zoonotic in nature and originate from wildlife reservoirs. Borna disease, caused by Borna disease virus 1 (BoDV-1), is an infectious disease affecting mammals, but recently it has also been shown to cause fatal encephalitis in humans. The endemic character of Borna disease points towards a nature-bound reservoir, with only one shrew species identified as reservoir host to date. Bats have been identified as reservoirs of a variety of zoonotic infectious agents. Endogenous borna-like elements in the genome of certain bat species additionally point towards co-evolution of bats with bornaviruses and therefore raise the question whether bats could serve as a potential reservoir of orthobornaviruses.

**Methods:**

Frozen brain samples (*n* = 257) of bats of seven different genera from Germany were investigated by orthobornaviral RT-PCR. Additionally, tissue slides of formalin-fixed paraffin-embedded material of a subset of these bats (*n* = 140) were investigated for orthobornaviral phosphoprotein by immunohistochemistry.

**Results:**

The brain samples were tested by RT-PCR without any evidence of orthobornavirus specific amplicons. Immunohistochemistry revealed a faint immunoreaction in 3/140 bats but with an untypical staining pattern for viral antigen.

**Conclusions:**

RT-PCR-screening showed no evidence for orthobornaviral RNA in the investigated bats. However, immunohistochemistry results should be investigated further to elucidate whether the reaction might be associated with expressed endogenous bornaviral elements or other so far unknown bornaviruses.

## Background

The increasing incidence of emerging infectious diseases (EID) represents a threat to public health. Interestingly, the majority of these EIDs are zoonotic in nature and originate from wildlife reservoirs. Due to their biological characteristics, particularly bats have been identified as reservoirs for many emerging viruses [1].

Many of these emerging viruses are RNA-viruses of the order *Mononegavirales*. In this order, the virus family *Bornaviridae* has been growing remarkably during recent years due to the discovery of several new species and genera. As of 2019, the taxonomy comprises three genera: *Carbovirus*, *Cultervirus* and *Orthobornavirus* [2]. Of the genus *Orthobornavirus*, two species, *Mammalian 1 orthobornavirus* and *Mammalian 2 orthobornavirus*, are known to affect mammals. Belonging to *Mammalian 1 orthobornavirus*, Borna disease virus 1 (BoDV-1) is still the most prevalent bornavirus in mammals. BoDV-1 is well known to cause severe and fatal neurological Borna disease in a variety of mammals, mainly horses and sheep. Recently BoDV-1 was identified as causal agent in fatal human encephalitis cases [3, 4]. This underlines the need to unravel potential sources of infection in order to prevent further animal and human cases, especially since no curative therapy or vaccination exists [3, 4].

Endemic areas for Borna disease are located in central Europe, such as Bavaria, Saxony-Anhalt and Saxony in Germany, St. Gallen and Canton of Grisons in Switzerland and Vorarlberg and Upper Austria in Austria [5, 6]. Phylogeny of BoDV-1 isolates reflects their geographical origin and respective endemic regions regardless of the host species they have been isolated from [5]. The endemic character, strong conservation of the viral genome and seasonal occurrence of the disease already pointed to a potential wildlife reservoir [7]. As the sequences of BD cases from neighbouring locations are particularly stable even over years, this wildlife reservoir was assumed to be territorially bound [5]. In several of the endemic areas, the bicolored white-toothed shrew (*Crocidura leucodon*), belonging to the order of Eulipotyphla, has been identified as natural reservoir of BoDV-1 [6, 8, 9]. However, beside the bicolored white-toothed shrew, no other reservoir, neither Eulipotyphla nor rodent, has been found yet. Bank voles have been experimentally proven to be susceptible to BoDV-1 [10] and serum antibodies against bornaviruses have been detected in free ranging bank voles [11]. Nevertheless, there is no evidence for naturally infected bank voles in endemic areas to date. Serological data have shown that several other free ranging small mammals, mostly belonging to the order of Rodentia, can also exhibit serum antibodies against *Bornaviridae*, but without any other evidence of BoDV-1-infection [9]. Only bicolored white-toothed shrews display a disseminated virus distribution and harbour BoDV-1 in excretory and secretory organs [9, 12] and shed infectious virus which suggests that they can transmit BoDV-1 [13].

Although several rodents and other small mammals are known as important reservoirs for many viruses, bats (order: Chiroptera) represent the vast majority of identified natural reservoirs of several virus families/species to date [1, 14]. For example, bats are known reservoirs for a growing list of RNA viruses, including rabies virus and other lyssaviruses, henipaviruses, coronaviruses and ebola virus [15]. Virus infections in bats follow the typical pattern of reservoirs with a persistent course lacking clinical disease [1, 16]. Several biological characteristics including gregariousness with large colonies, seasonal migrating and long life span make bats suitable to carry and spread viruses [16]. Seasonal migrating and wide hunting territories of bats can lead to wide distribution of harboured viruses [1], however, shedding in pulses and additional local factors can lead to local transmission events [16]. In Europe, 35 bat species from the order Chiroptera can be found [17]. These European bats share the same biological characteristics, but transmission events of zoonotic viruses are rare due to smaller dimensions of populations and colonies [18]. In Germany, 25 bat species can be encountered from the family Rhinolophidae and Vespertilionidae [19]. They include common native species, such as members of the genera *Pipistrellus*, *Myotis* and *Vespertilio*, and some seriously endangered species like *Rhinolophus hipposideros*, *Myotis emarginatus* and *Barbastella barbastellus* [19]. Several bat species, for example *Myotis natteri*, use roosts in barns and stables [20], which facilitates the possibility of virus transmission to livestock [18].

In addition, endogenous bornavirus-like elements (EBL) have been detected in the genome of several bat species [21–24]. These EBL are DNA-sequences in bat genomes displaying considerable sequence identities to present-day bornaviral genes on amino acid level of about 30–50% [25]. In some bat species, transcription of EBL similar to the bornaviral RNA-dependent RNA-polymerase has been reported [22]. Further analysis of these EBL strongly hint at ancestral and repeated contact between bats and bornaviruses during their evolution at least 11,8 million years ago [23, 26]. The function of these EBL is still discussed and an immunological benefit in the interaction with bornaviruses has been suggested in some mammalian species [22, 27]. As the retaining of the expressed EBL in the bat genome despite evolutionary selection requires resources, a benefit of the EBL and regular encounters between bornaviruses and bats during evolution seem plausible [22].

In conclusion, due to the continuous detection of new viruses in bats, the unclear situation regarding additional potential BoDV-1-reservoirs and molecular evidence for co-evolution of bats and bornaviruses, this study was conducted to investigate the potential presence of the most common orthobornaviruses in bats from endemic and non-endemic areas in Germany.

## Methods

Two hundred fifty-seven brain samples of bats from Germany (97 from endemic regions in Bavaria) were provided by the Leibniz Institute for Zoo and Wildlife Research (Leibniz-IZW), Berlin (79 *Pipistrellus sp.*, 67 *Nyctalus sp.*, 57 *Myotis sp.*, 22 *Eptesicus sp.*, 17 *Vespertilio sp.*, 12 *Plecotus sp.*, 2 *Barbastella sp.*, 1 bat without species identification), and stored frozen at − 80 °C. Additionally, bat organs from diagnostic necropsies archived as formalin-fixed paraffin-embedded (FFPE) material of 101 bats from the German federal state of Bavaria (31 *Pipistrellus sp.*, 14 *Vespertilio sp.*, 12 *Eptesicus sp.*, 12 *Nyctalus sp.*, 10 *Plecotus sp*., 8 *Myotes sp*.) and 39 bats from the federal state of Hesse (5 *Myotis sp*., 2 *Pipistrellus sp*., 2 *Plecotus sp*., 1 *Nyctalus sp*., 29 bats without species identification) were provided by the Leibniz-IZW, the State Veterinary Institute of Giessen (Landesbetrieb Hessisches Landeslabor) and the Department of Animal Ecology and Systematics, Giessen. All organ tissues were retrieved from diagnostic necropsy material from carcasses submitted by bat rehabilitation centres and bat researchers in Germany to the respective institution. Samples from the Leibniz-IZW were archived materials from a previous larger study on disease and causes of death in European bats from Germany [28].

For screening for orthobornaviral RNA (200 bp of X/P-ORF), brain samples were analysed by a two-step RT-PCR detecting a broad spectrum of orthobornaviruses (see below). RNA isolation and RT-reaction was performed with RNEasy Mini kit (Qiagen) and Quantitect Reverse Transcription Kit (Qiagen) according to manufacturer’s instructions, respectively. PCR was performed with MyTaq HSMix (Bioline) under manufacturer’s standard condition with degenerated primers (Additional file 2: Table S1) [29]. These primers were designed to detect viruses of seven species of the genus *Orthobornavirus* (*Mammalian 1 orthobornavirus*, *Mammalian 2 orthobornavirus*, *Passeriform 1 orthobornavirus*, *Passeriform 2 orthobornavirus*, *Psittaciform 1 orthobornavirus*, *Psittaciform 2 orthobornavirus* and *Waterbird 1 orthobornavirus*), but not viruses of the species *Elapid 1 orthobornavirus* of the genus *Orthobornavirus* or viruses of the genera *Carbovirus* or *Cultervirus*. The applied RT-PCR assay has been proven to detect several known pathogenic members of the genus *Orthobornavirus* (BoDV-1, variegated squirrel bornavirus 1 (VSBV-1), parrot bornavirus 2 (PaBV-2), parrot bornavirus 4 (PaBV-4)) [29]. As internal control, glyceraldehyde-3-phosphate-dehydrogenase-(GAPDH)-amplification (402 bp) was included. As positive control, isolated RNA from a BoDV-1-positive mouse was used, and a formerly negatively tested bat served as negative control. Lengths of amplicons were visualized with gel electrophoresis (2% agarose gel with 3% Midori Green (Biozym)) according to manufacturer’s instructions and commercial Sanger sequencing of orthobornaviral amplicons was performed for positive controls (GATC, Eurofins Genomics). BoDV-1 negatively-tested bat-RNA was spiked with serial dilutions of either BoDV-1-RNA, VSBV-1-RNA, PaBV-2-RNA or PaBV-4-RNA to assess specificity and sensitivity.

To screen for bornaviral antigen, immunohistochemistry was performed using a polyclonal antibody for the detection of bornaviral phosphoprotein (antibody p24). This antibody is known for its cross-reactivity also with the phosphoprotein of PaBV-2 and PaBV- 4 of the species *Psittaciform 1 orthobornaviruses* [30] and VSBV-1 [31]. All reactions were compared to a negative control slide incubated with a rabbit serum (Rabbit Immunoglobulin Fraction, Dako). Organs with positive immunostaining were further examined with a panel of antibodies to examine specificity of this reaction. The panel included two antibodies directed against the viral nucleoprotein of BoDV-1 (monoclonal antibody Bo18 [32] and polyclonal antibody anti-BoDV-N [4]) and a mix of polyclonal antibodies detecting VSBV-1-nucleoprotein and phosphoprotein [provided by Dennis Tappe, Bernhard Nocht Institute Hamburg]. To exclude unspecific reaction of the polyclonal rabbit-antibodies, a polyclonal antibody detecting rabies virus as well as a second control rabbit serum (Thermofisher) were used as additional negative controls (details on immunohistochemistry protocols in Additional file 4: Table S2).

## Results

By RT-PCR-screening, in 239/257 samples GAPDH-amplicons could be obtained, the other 19 samples were excluded due to insufficient quality. These 239 samples were tested for orthobornaviral RNA and no specific amplicons regardless of origin from endemic or non-endemic areas were observed. The control consisting of RNA of a BoDV-1 infected mouse was correctly amplified as verified by correct size on the gel and respective sequences (Additional file 1: Figure S1). Spiking of bat RNA with serial dilutions of various orthobornavirus-RNA demonstrated the detection limit of 5000 orthobornavirus copies in 660 ng RNA.

By immunohistochemistry applying the polyclonal antibody p24 specific for the phosphoprotein, a faint reaction was found in 3/140 animals, in particular located in the cytoplasm of smooth muscle cells of the intestine. All respective negative control slides were without any immunoreaction regardless which control antibody was used. No immunoreactivity was found using the monoclonal antibody Bo18 specific for the BoDV-1-nucleoprotein in these samples. Immunoreactivity was found using the polyclonal antibody detecting BoDV-1-nucleoprotein in one sample and using the polyclonal antibodies detecting VSBV-nucleoprotein and phosphoprotein in two samples. However, in 17/140 other animals a comparable immunoreaction was observed in negative control slides using control rabbit serum or polyclonal anti-rabies antibodies (details in Additional file 5: Table S3 and Additional file 3: Figure S2).

## Discussion

Recent cases of fatal encephalitis in humans due to BoDV-1 infection strengthen the need to survey potential wildlife reservoirs and identify potential risk factors for infection. Although the bicolored white-toothed shrew has been identified as indigenous reservoir of BoDV-1, other potential reservoirs or animal carriers are still unknown so that further investigations of small mammals including bat species are urgently needed. Bats have already been discovered as reservoir of emerging and highly pathogenic viruses. Many factors, such as their gregarious way of life, can facilitate pathogen transmission to other bats and virus persistence in the population. In European bats, only few zoonotic viruses have been discovered [18, 33, 34] and the overall hazard for humans is comparably low [18]. As some bats take roosts in barns and stables [20] and bat carcasses are found in close proximity to agriculture [35], a risk of sporadic transmission events to livestock animals can be assumed if viruses can be detected. Animal movement across borders of endemic regions during hunting and migration of bats seems to contradict stable geographical clustering of BoDV-1 isolates and the hypothesis of a territorially bound reservoir [5]. Nonetheless, consistent usage of the same roosts as summer or winter quarter may support observed clustering and could facilitate rare endemic transmission. Additionally, the molecular evidence for co-evolution of bats and bornaviruses [22, 27] could suggest the possibility of infections of other potentially so far unknown bornaviruses beside the tested orthobornaviruses. Therefore, this study aimed to examine the possible role of bats as carrier and reservoir of orthobornaviruses such as BoDV-1 as one of the most common virus.

All samples originated from bats which died because of injuries or disease. They were part of a previous study on diseases and causes of death in European bats, where traumatic injuries and inflammatory lesions, partly due to bacterial infections, were the major cause of deaths in these animals [28]. Since Borna disease in animals is known to be endemic in specific areas of Germany [5], samples were sorted by regional origin corresponding to known endemic regions (Bavaria) and other non-endemic regions in Germany. However, as some bat species tend to have wide hunting territories or migrate during the year and can cross the borders of endemic regions, this sorting might bear a risk of bias. The study includes samples from available bat species and is not limited to bat species suspected to interact with bornaviruses [23, 24] as interspecies virus transmission has already been observed [1, 14].

Interestingly, the screening by RT-PCR for orthobornaviral RNA provided no evidence of orthobornavirus infection in the investigated bats. The detection limit of the applied orthobornavirus RT-PCR was 0.01 ng/μl orthobornaviral RNA in 700 ng/μl mammalian RNA according to literature [29] and 5000 copies in 660 ng RNA in our own testing. Therefore, already low amounts of viral RNA should have been detected as verified by the correct amplification of all control orthobornavirus species. Spillover host [36] and reservoir species [12] regularly yield high amount of viral RNA, much higher than the detection level of the applied RT-PCR assay. However, the presence of previously undiscovered bornavirus species, such as the ones recently described in reptiles and classified as carboviruses [37] cannot be excluded and could be further investigated by broad and undirected approaches, such as metagenomics.

In contrast to the RT-PCR results, the faint immunohistochemical reaction in smooth muscles of three animals raises the question whether an antigen with cross-reactivity or a bornaviral phosphoprotein is present. However, in several other animals similar reactions were detected applying unrelated polyclonal antibodies produced in rabbits and unspecific rabbit serum. Moreover, the staining pattern is rather untypical for BoDV-1 but occurs regularly in avian bornavirus infections [30]. The immunohistochemical reaction was not observed using a monoclonal antibody against the nucleoprotein of BoDV-1. It was observed in one animal using a polyclonal antibody against the nucleoprotein of BoDV-1 and in two animals using polyclonal antibodies against proteins of variegated squirrel bornavirus-1. Thus, an infection with another, so far unknown bornavirus could not completely be excluded and has to be investigated in further studies. Scarcity and limited quality of material impeded further immunohistochemical and molecular investigations. As endogenous bornaviral elements similar to bornaviral RNA-dependent RNA-polymerase have been found in bats [22], a translated endogenous element could also have been detected by immunohistochemistry due to cross-reactivity. However, an endogenous bornaviral-phosphoprotein-like protein has not been found yet and some authors have discussed a deleterious effect of endogenization of bornaviral phosphoprotein [38]. On the contrary, an endogenous bornaviral-P-like protein might also help prevent against bornavirus infection as the bornaviral polymerase is inhibited by a disturbed nucleoprotein-phosphoprotein-ration [39].

## Conclusions

To summarize, RT-PCR-screening of tissues from European bats revealed no evidence for orthobornaviral RNA. Further studies could unravel whether the immunohistochemical reactions might be due to expression of endogenous sequences gained during evolution of the bat species or even new bornaviruses.

## Supplementary information

**Additional file 1: Figure S1.** Gel electrophoresis of PCR Products. 1–10: Borna-negative samples of sufficient quality with GAPDH-band at 402 bp-amplicon length; 11: Sample of insufficient quality without GAPDH-band; NTRT: No template reverse transcription-reaction control; PC: Positive control (BoDV-1-positive mouse); NC: Negative control (BoDV-1 negative bat); NTC: No template control of PCR; bp: base pairs.

**Additional file 2: Table S1.** Primer Sequences. Information about primers.

**Additional file 3: Figure S2.** Immunohistochemistry reactions in the intestine of *Eptesicus nilssonii.* 2A polyclonal antibody p24 specific for orthobornavirus phosphoprotein, staining of smooth muscle cells; 2B polyclonal antibody anti-BoDV-N specific for BoDV-1 nucleoprotein, no immunoreaction; 2C polyclonal antibodies VSBV-N and VSBV-P specific for VSBV-1 nucleoprotein and phosphoprotein, faint staining of smooth muscle cells; 2D negative control: rabbit immunoglobulin fraction, no immunoreaction; 2E negative control: control rabbit serum, no immunoreaction; 2F negative control: polyclonal antibody specific for rabies virus, no immunoreaction.

**Additional file 4: Table S2.** Immunhistochemistry details. Information about antibodies used in the immunohistochemistry.

**Additional file 5: Table S3.** Bats with positive immunoreactivity. Information about all bats with immunoreaction with antibody p24.

## Data Availability

All data generated or analysed during this study are included in this published article and its supplementary information files.

## Abbreviations

BoDV-1: Borna disease virus 1
EBL: Endogenous bornavirus-like elements
EID: Emerging infectious disease
FFPE: Formalin-fixed paraffin-embedded
GAPDH: Glyceraldehyde-3-phosphate-dehydrogenase
PaBV-2: Parrot bornavirus 2
PaBV-4: Parrat bornavirus 4
sp.: Species
VSBV-1: Variegated squirrel bornavirus 1
