# Supplementary figures and images for: No evidence for European bats serving as reservoir for Borna disease virus 1 or other known mammalian orthobornaviruses
Source: Virol J. 2020 Jan 30;17:11. doi: 10.1186/s12985-020-1289-3 (PMC6993374; doi:10.1186/s12985-020-1289-3)

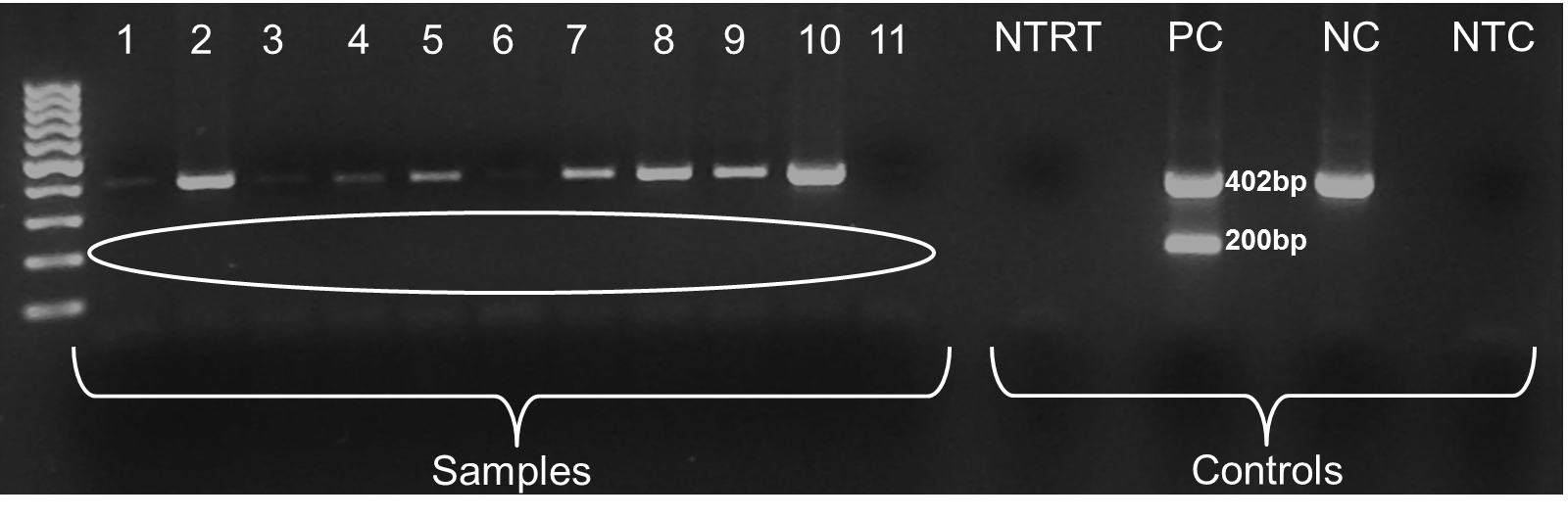

Supplement: Supplementary file 1 — Additional file 1: Figure S1. Gel electrophoresis of PCR Products. 1–10: Borna-negative samples of sufficient quality with GAPDH-band at 402 bp-amplicon length; 11: Sample of insufficient quality without GAPDH-band; NTRT: No template reverse transcription-reaction control; PC: Positive control (BoDV-1-positive mouse); NC: Negative control (BoDV-1 negative bat); NTC: No template control of PCR; bp: base pairs. [file 12985_2020_1289_MOESM1_ESM.tif]
