# Supplementary material for: No evidence for European bats serving as reservoir for Borna disease virus 1 or other known mammalian orthobornaviruses
Source: Virol J. 2020 Jan 30;17:11. doi: 10.1186/s12985-020-1289-3 (PMC6993374; doi:10.1186/s12985-020-1289-3)
Supplement: Supplementary file 2 — Additional file 2: Table S1. Primer Sequences. Information about primers. [file 12985_2020_1289_MOESM2_ESM.docx]

Additional Table 1: Primer sequences

| **Primer** | **Sequence (5‘-3‘)** | **Product** |
| --- | --- | --- |
| OrthoBorna Forward | CGCGACCMTCGAGYCTRGT | 200bp  [KF275184.1] |
| OrthoBorna Reverse | GACARCTGYTCCCTTCCKGT |  |
| GAPDH Forward | GGTGATGCTGGTGCTGAGTAT | 402bp  [NM_017008.4] |
| GAPDH Reverse | GGATGACCTTGCCCACAG |  |
