# Supplementary material for: No evidence for European bats serving as reservoir for Borna disease virus 1 or other known mammalian orthobornaviruses
Source: Virol J. 2020 Jan 30;17:11. doi: 10.1186/s12985-020-1289-3 (PMC6993374; doi:10.1186/s12985-020-1289-3)
Supplement: Supplementary file 4 — Additional file 4: Table S2. Immunhistochemistry details. Information about antibodies used in the immunohistochemistry. [file 12985_2020_1289_MOESM4_ESM.docx]

Additional Table 2: Immunohistochemistry details

| **Antibody** | **Dilution** | **Pretreatment** | **Detection system** | **Origin** |
| --- | --- | --- | --- | --- |
| polyclonal rabbit-anti-p24 (p24) | 1:2000 | none | biotinylated anti-rabbit IgG+ ABC-method | Prof. Garten, Marburg, Germany |
| monoclonal mouse-anti-p38 (Bo18) | 1:500 | none | biotinylated anti-mouse IgG + ABC-method | Dr. Herzog, Giessen, Germany |
| polyclonal rabbit-anti-BoDV-N | 1:2000 | none | biotinylated anti-mouse IgG + ABC-method | Prof. Dennis Tappe, Hamburg, Germany |
| polyclonal rabbit-anti-VSBV-N + polyclonal rabbit-anti-VSBV-P | 1:2000 | none | biotinylated anti-mouse IgG + ABC-method | Prof. Dennis Tappe, Hamburg, Germany |
| rabbit immunoglobulin fraction | 1:2000 | none | biotinylated anti-mouse IgG + ABC-method | Dako, Agilent |
| control rabbit serum | 1:2000 | none | biotinylated anti-mouse IgG + ABC-method | Thermofisher |
| polyclonal rabbit-anti-rabies | 1:2000 | none | biotinylated anti-mouse IgG + ABC-method | Dako, Agilent |
