# Supplementary material for: No evidence for European bats serving as reservoir for Borna disease virus 1 or other known mammalian orthobornaviruses
Source: Virol J. 2020 Jan 30;17:11. doi: 10.1186/s12985-020-1289-3 (PMC6993374; doi:10.1186/s12985-020-1289-3)
Supplement: Supplementary file 5 — Additional file 5: Table S3. Bats with positive immunoreactivity. Information about all bats with immunoreaction with antibody p24. [file 12985_2020_1289_MOESM5_ESM.docx]

Additional Table 3: Bats with positive immunoreactivity

| **Species** | **Origin** | **PCR-result (brain)** | **Immunohistochemistry** | | | | | | | | |
| --- | --- | --- | --- | --- | --- | --- | --- | --- | --- | --- | --- |
|  |  |  | **Tissues without immunoreaction** | **Tissues with immunoreaction** | **polyclonal anti-BoDV-P (p24)** | **monoclonal anti-BoDV-N (Bo18)** | **polyclonal anti-BoDV-N** | **polyclonal anti-VSBV-N/-P** | **rabbit immunoglobulin fraction** | **control rabbit serum** | **polyclonal anti-rabies** |
| **Animals with no immunoreaction in control slides** | | | | | | | | | | | |
| *Vespertilio murinus* | Bavaria | negative | brain, tongue, trachea, lung, salivary gland, larynx | intestine/smooth muscle | positive | negative | positive | positive | negative | negative | negative |
| *Eptesicus nilssonii* | Bavaria | negative | brain, liver, trachea, lymph node, liver | intestine/smooth muscle | positive | negative | negative | positive | negative | negative | negative |
| without species identification | Hesse | no material available | lung, heart, spleen, liver, lymph node, skeletal muscle, salivary gland | intestine/smooth muscle | positive | negative | negative | n/a | negative | negative | n/a |
| **Animals with immunoreaction in control slides** | | | | | | | | | | | |
| *Pipistrellus kuhlii* | Bavaria | negative | brain, heart, tongue | trachea/paratracheal connective tissue; lung/alveolar cells | positive | negative | positive | positive | negative | positive | negative |
| *Vespertilio murinus* | Bavaria | negative | brain, larynx, spleen, salivary gland | lung/alveolar cells | positive | negative | positive | positive | negative | positive | negative |
| *Pipistrellus pipistrellus* | Bavaria | no material available | brain, testis, intestine, salivary gland, lung, trachea | tongue/superficial epithelial cells | positive | negative | positive | positive | negative | positive | positive |
| *Pipistrellus pipistrellus* | Bavaria | excluded due to insufficient quality | brain, tongue, pancreas, larynx, salivary gland, intestine | lung/alveolar cells | positive | negative | positive | positive | negative | positive | positive |
| *Plecotus auritus* | Bavaria | negative | brain, testis, lung, intestine | tongue/superficial epithelial cells | positive | negative | positive | positive | negative | positive | positive |
| *Pipistrellus pipistrellus* | Bavaria | excluded due to insufficient quality | brain, intestine | trachea/paratracheal connective tissue; salivary gland/secret | positive | negative | positive | positive | negative | positive | positive |
| *Eptesicus serotinus* | Bavaria | negativ | brain, tongue, lung, salivary gland, larynx | intestine/smooth muscle | positive | negative | positive | positive | negative | positive | positive |
| *Pipistrellus kuhlii* | Bavaria | excluded due to insufficient quality | brain, tongue, salivary gland, larynx | intestine/smooth muscle; lung/alveolar cells | positive | negative | positive | positive | negative | positive | positive |
| *Myotis mystacinus* | Bavaria | excluded due to insufficient quality | brain, lung, salivary gland, trachea, tongue, testis | intestine/smooth muscle | positive | negative | negative | positive | negative | positive | positive |
| *Pipistrellus pipistrellus* | Bavaria | excluded due to insufficient quality | brain, heart, lung, esophagus, lung, skeletal muscle | intestine/smooth muscle | positive | negative | positive | positive | negative | positive | positive |
| *Eptesicus nilssonii* | Bavaria | negative | brain, lung | intestine/smooth muscle | positive | negative | positive | positive | negative | positive | positive |
| *Myotis mystacinus* | Bavaria | negative | brain, skeletal muscle, | lung/alveolar cells; salivary glands/secretion | positive | negative | positive | positive | negative | positive | positive |
| *Pipistrellus pipistrellus* | Bavaria | excluded due to insufficient quality | brain, heart, salivary gland, skeletal muscle | Intestine/smooth muscle | positive | negative | positive | positive | negative | positive | positive |
| *Eptesicus nilssonii* | Bavaria | negativ | brain, heart, skeletal muscle | intestine/smooth muscle | positive | negative | n/a | n/a | negative | n/a | n/a |
| without species identification | Hesse | no material available | brain, skin, heart, lung, trachea, salivary gland | intestine/smooth muscle | positive | negative | positive | n/a | negative | positive | n/a |
| without species identification | Hesse | no material available | lung, heart, kidney, tongue, liver | intestine/smooth muscle | positive | negative | positive | negative | negative | positive | negative |
| without species identification | Hesse | no material available | heart, liver, brain | intestine/smooth muscle | positive | negative | negative | positive | negative | positive | negative |
